# Ice-templated Self-assembly of VOPO_4_–Graphene Nanocomposites for Vertically Porous 3D Supercapacitor Electrodes

**DOI:** 10.1038/srep13696

**Published:** 2015-09-03

**Authors:** Kwang Hoon Lee, Young-Woo Lee, Seung Woo Lee, Jeong Sook Ha, Sang-Soo Lee, Jeong Gon Son

**Affiliations:** 1 Photo-Electronic Hybrids Research Center, Korea Institute of Science and Technology, Seoul 136-791, Republic of Korea; 2 Department of Engineering Science, University of Oxford, Oxford OX1 3PJ, United Kingdom; 3 George W. Woodruff School of Mechanical Engineering, Georgia Institute of Technology, Atlanta, GA 30332, USA; 4 Department of Chemical and Biological Engineering, Korea University, Seoul 136-701, Republic of Korea; 5 KU-KIST Graduate School of Converging Science and Technology, Korea University, Seoul 136-701, Republic of Korea

## Abstract

A simple ice-templated self-assembly process is used to prepare a three-dimensional (3D) and vertically porous nanocomposite of layered vanadium phosphates (VOPO_4_) and graphene nanosheets with high surface area and high electrical conductivity. The resulting 3D VOPO_4_–graphene nanocomposite has a much higher capacitance of 527.9 F g^−1^ at a current density of 0.5 A g^−1^, compared with ~247 F g^−1^ of simple 3D VOPO_4_, with solid cycling stability. The enhanced pseudocapacitive behavior mainly originates from vertically porous structures from directionally grown ice crystals and simultaneously inducing radial segregation and forming inter-stacked structures of VOPO_4_–graphene nanosheets. This VOPO_4_–graphene nanocomposite electrode exhibits high surface area, vertically porous structure to the separator, structural stability from interstacked structure and high electrical conductivity, which would provide the short diffusion paths of electrolyte ions and fast transportation of charges within the conductive frameworks. In addition, an asymmetric supercapacitor (ASC) is fabricated by using vertically porous VOPO_4_–graphene as the positive electrode and vertically porous 3D graphene as the negative electrode; it exhibits a wide cell voltage of 1.6 V and a largely enhanced energy density of 108 Wh kg^−1^.

Supercapacitors have unique advantages over lithium-ion batteries in terms of high power delivery and long cycling life, and they are emerging as attractive electrochemical energy-storage devices for future energy-storage application^1^,^2^. However, supercapacitors usually have lower energy density than lithium-ion batteries. Intensive research efforts have been devoted to the enhancement of their energy density while retaining their intrinsic high power density^3^,^4^. Supercapacitors can be divided into two types based on the charge-storage mechanism: double-layer capacitance and pseudocapacitance^5^. Since the charges in electrochemical double-layer capacitors (EDLCs) are stored on the electrode surface, carbon materials such as activated carbons or newly developed carbon nanotubes and graphene have been selected owing to their large specific surface area, high electrical conductivity, and chemical stability^6^,^7^. However, electrodes based on two-dimensional (2D) graphene often suffer from irreversible sheet-to-sheet restacking due to the strong interlayer van der Waals force. This restacking phenomenon severely decreases the surface area and disturbs ion diffusion in electrolyte, resulting in unsatisfactory capacitive performance. Therefore, several methods for preventing aggregation have been developed, which include adding spacers^8^, employing a microparticle template^4^, adding hydrothermal treatment^9^, and using crumpled graphene^10^. And recently, vertically aligned graphene sheet structures for fast ion diffusion was also realized using simple hand-rolling and cutting processes and showed efficient electrochemical characteristics even at a high scan rate^11^.

The pseudocapacitors have fast surface redox reactions on metal oxides or conducting polymers, exhibiting much higher energy density than EDLCs^6^,^12^. Transition-metal oxides, such as MnO_2_, Co(OH)_2_, NiO, Ni(OH)_2_, and V_2_O_5_, have been widely utilized as pseudocapacitive electrode materials^3^,^13^,^14^,^15^,^16^,^17^. However, the intrinsic high electrical resistances of metal oxides limit their charge and discharge rate and the power density of the electrodes, which are essential factors for high-power applications. To overcome low electrical conductivity of metal oxides, many researchers have investigated the porous nano-structures^18^ or composite structures with high-conductivity carbon materials^19^. However, issues concerning random agglomeration and uncontrollable distribution of inorganic nanoparticles on the high-conductivity carbon surface remain unresolved. Therefore, a new strategy for improving the conductivity and maintaining the high surface area of the metal oxide is required to maximize the number of active sites for pseudocapacitance with lower charge-transfer resistance.

Among the high-capacitance oxide materials, layered vanadium phosphates (VOPO_4_) has been intensively studied as an electrode material of lithium-ion battery because of their multiple oxidation states (from V^0^ to V^5+^) and high theoretical specific capacity (827 mA h g^−1^)^20^,^21^,^22^. However, it is seldom used in pseudocapacitors because VOPO_4_ also has an intrinsic high electrical resistance (~3.0 × 10^7^ Ω cm^−1^) and the layered structure of bulk VOPO_4_ has limited surface area, which lowers the power density of electrochemical devices. Recently, Wu and coworkers demonstrated the decent performance of a vacuum-filtered VOPO_4_–graphene thin film as a supercapacitor in thin-film geometry^23^. However, the VOPO_4_–graphene thin film electrode also has a structural handicap that stacked 2D materials has less active surface area and significantly meanders the ion diffusion path of the electrolyte^11^. In this context, we noticed ice-templated method, which use a directional growth of ice crystals as a template, for the fabricating vertically porous three-dimensional (3D) structures of polymers^24^, nanoparticles^18^,^25^ and super-elasticity graphene^26^. However, so far, this approach has been rarely adopted for carbon based energy-storage systems.

In this work, we used the ice-templated self-assembly technique to realize vertically porous 3D microstructures of interstacked VOPO_4_–graphene nanocomposites in order to attain superior performance of supercapacitor electrodes. The ice-templated self-assembly was a very efficient approach to maximize the performance of supercapacitor electrodes because it formed micrometer-sized, vertically porous structures from directionally grown ice crystals and simultaneously induced radial segregation in the suspension containing VOPO_4_–graphene nanosheets resulting in interstacked 3D VOPO_4_–graphene nanostructures with a highly conductive framework. The vertically porous 3D structure of the graphene-based framework also had a high surface area and vertical microchannels as a highway of ion transport in the electrolyte. At the same time, the interstacked structures of 3D VOPO_4_–graphene were strongly bound by hydrogen bonding between VOPO_4_ and graphene^23^, and the maximized plane contact between the sheets brought about the fluent charge transfer along the shortest transport pathways^27^ and structural stability during multiple redox reactions^28^. Based on the 3D VOPO_4_–graphene electrode, we fabricated a coin-shaped asymmetric supercapacitor (ASC) consisting of vertically porous 3D VOPO_4_–graphene as the positive electrode and vertically porous 3D graphene as the negative electrode (referred to as VP3D-VOPO_4_–RGO/VP3D-RGO ASC hereafter) without any binders, and the ASC exhibited a high energy density of 108 Wh kg^−1^.

## Results and Discussion

**Figure 1:**
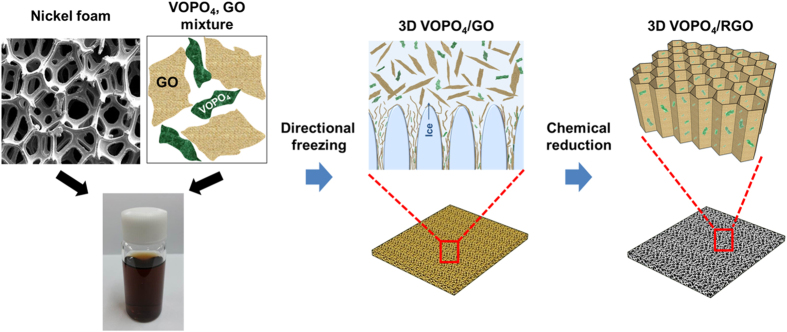
Schematic illustrating the construction of the VOPO_4_–RGO nanocomposite with vertically porous 3D structures through ice-templated self-assembly.

**Figure 2:**
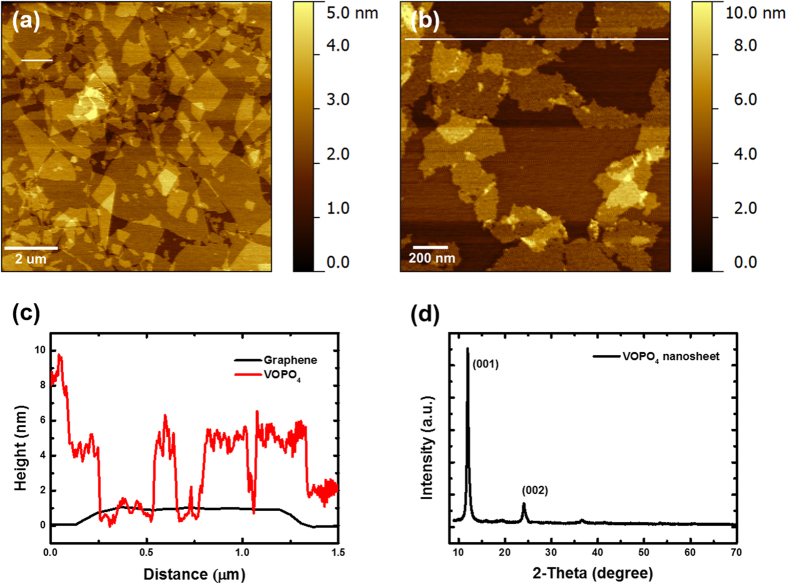
AFM images of spin-cast (a) graphene oxide (GO) and (b) VOPO_4_ nanosheets on freshly cleaved mica. (**c**) Corresponding AFM height profiles of GO and VOPO_4_ nanosheets. (**d**) XRD pattern of pure 2D VOPO_4_ nanosheets.

Figure 1 illustrates the preparation procedure for the fabrication of vertically porous 3D nanocomposites consisting of VOPO_4_ and reduced graphene oxide (RGO). First, VOPO_4_ nanosheets were synthesized from vanadium oxide powder and phosphoric acid; the prepared nanosheets were then exfoliated by sonication in isopropanol^29^. The graphene oxide (GO) was produced by the modified Hummers method from graphite flakes^30^. The morphology and size of the exfoliated nanosheets were examined by atomic force microscopy (AFM) in the tapping mode, as shown in Fig. 2. The average lateral area of the GO nanosheets were approximately 6 μm^2^ and they had an average thickness of ~1 nm, indicative of the successful exfoliation of each nanosheet to a monolayer (Fig. 2a,c)^31^. The VOPO_4_ nanosheets showed smaller lateral sizes than the GO nanosheets, with average area of ~0.4 μm^2^ (Fig. 2b). The thickness of the VOPO_4_ nanosheet was about 5 nm, as shown in Fig. 2c, which indicates that the exfoliated nanosheets consisted of 6–7 layers because the layer spacing of the VOPO_4_∙2H_2_O lattice structure has a known value of 7.41 Å^32^. To confirm the successful synthesis and crystal structure of the VOPO_4_ nanosheets, we performed X-ray diffraction (XRD) measurements on powder prepared from the nanosheets. As shown in Fig. 2d, the characteristic diffraction peaks of 11.8° (001), and 24.1° (002) are attributed to the tetragonal VOPO_4_·2H_2_O with layered structures (PCPDF card no. 84-0111)^23^.

Suspensions of GO nanosheets in water and VOPO_4_ nanosheets in isopropanol were mixed together in different ratios and sonicated for 30 min to obtain various homogeneous solutions. A typical 3D VOPO_4_–RGO nanocomposite electrode could be prepared by immersing 1-mm-thick nickel foam in a suspension of VOPO_4_ and GO. Using unidirectional freezing in liquid nitrogen, the VOPO_4_–GO mixture self-assembled according to a directionally grown ice template. Freeze-drying of the frozen samples were executed to acquire vertically porous 3D VOPO_4_–GO structures in nickel foam without any structural collapse. During the ice-templated self-assembly process, as the liquid suspension froze, the dispersed VOPO_4_ and GO nanosheets were expelled from the perpendicularly growing ice crystals and accumulated radially between them. This directional segregation promoted 2D plane–plane interactions, resulting in the directed self-assembly of VOPO_4_–GO nanosheets interstacked between the ice crystals, which is different from simple coagulation in hydrogel assembly. After hydrazine vapor treatment, the reduction of GO in 3D VOPO_4_–RGO nanocomposite was completed without chemical changes of VOPO_4_ sheets, which was confirmed by X-ray photoelectron spectroscopy (XPS) analysis and Raman spectroscopy in Figure S1 in supplementary information. This hybrid of VOPO_4_ and RGO exhibited an electrical conductivity of ~1.3 × 10^2^ Ω cm^−1^, which is five orders of magnitude higher than that of the pure VOPO_4_ nanosheets (~3.0 × 10^7^ Ω cm^−1^). In addition, nitrogen physisorption measurements revealed that the specific surface area of the 3D VOPO_4_–RGO nanocomposite had a noticeably high value of 325 m^2^ g^−1^ because of the porous structures.

**Figure 3:**
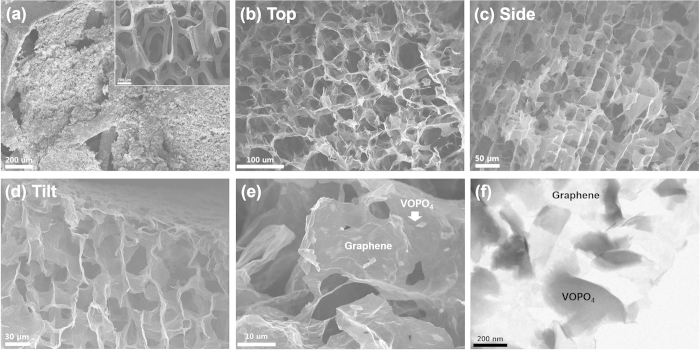
(**a**) SEM image of the porous 3D microstructures of the VOPO_4_–RGO nanocomposite developed in nickel foam; the inset shows the pristine nickel foam. (**b**) Top view, cross-sectional (**c**) side view and (**d**) tilt view of porous 3D microstructures of the VOPO_4_–RGO nanocomposite prepared via ice-templated self-assembly. High-magnification (**e**) SEM and (**f**) TEM images of the porous VOPO_4_–RGO nanocomposite.

Figure 3 shows the electrode, made of the ice-templated 3D VOPO_4_–RGO nanocomposite within nickel foam, as a current collector and a structural support. The 1:1 weight ratio of 3D VOPO_4_–RGO nanocomposite was successfully and completely incorporated into the nickel foam to form an interconnected 3D porous network, as can be seen in Fig. 3a. For the details of porous structures, Fig. 3b shows top-view of 3D VOPO_4_–RGO nanocomposite that several micrometer-sized pores are regularly packed like Voronoi diagram and the VOPO_4_–RGO composites are successfully constituted the framework. In the Fig. 3c,d, cross-sectional side and tilt images of the 3D VOPO_4_–RGO nanocomposite indicates that unidirectional freezing and growth of the close-packed ice crystals produced vertically aligned microchannel structures all the way through which were oriented along the freezing direction. Because this vertically aligned microchannel can be a highway of ion transport in the electrolyte, the vertically porous 3D structure of the VOPO_4_–RGO nanocomposite can allows rapid ion diffusion from/to the bulk electrolyte (separator)^11^. High-resolution scanning electron microscopy (SEM) and transmission electron microscopy (TEM) images (Fig. 3c,d, respectively) show that approximately 15 times larger RGO sheets mainly constitute the porous frameworks and smaller VOPO_4_ nanosheets clung to both sides of the RGO sheets to form a sandwich-like interstacked structure, which was also known to be tightly connected by hydrogen bonds^23^. This interstacked structure might represent the ideal geometry for electrochemical reactions because of the structural stability and the minimal contact resistance between VOPO_4_ and graphene. Thus, this 3D VOPO_4_–RGO nanocomposite had a highly favorable porous structure for application as a supercapacitor electrode material.

**Figure 4:**
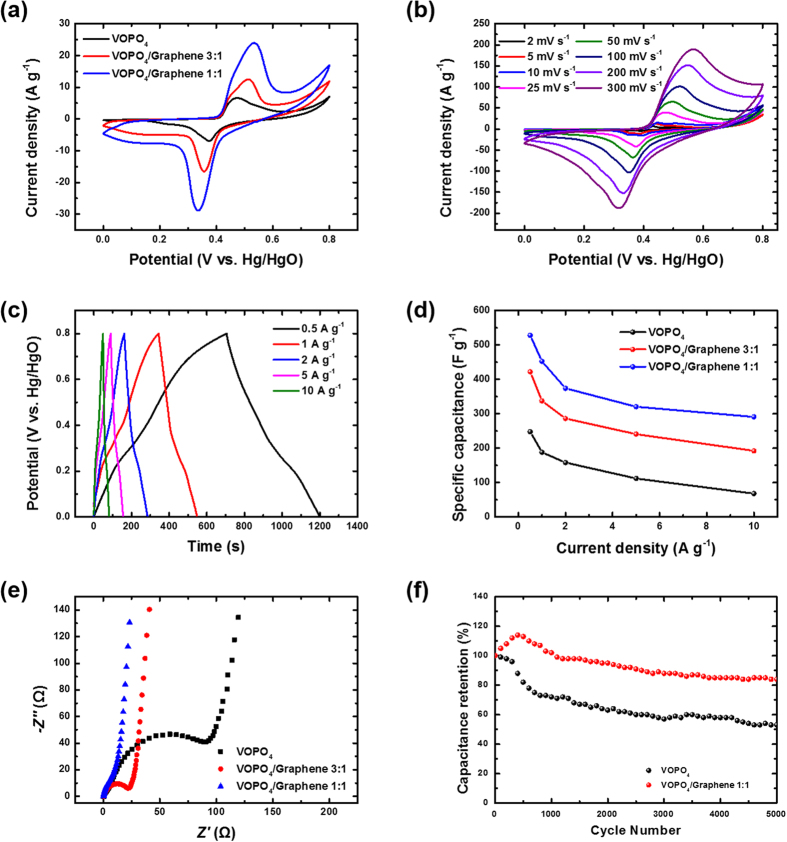
(**a**) CV curves of 3D VOPO_4_–RGO nanocomposite electrodes with varying mixing ratios and pristine VOPO_4_ electrode at a scan rate of 25 mV s^−1^ in 6 M KOH electrolyte. (**b**) CV curves of 1:1 VOPO_4_–RGO composite at various scan rates. (**c**) Galvanostatic charge–discharge curves of 1:1 VOPO_4_–RGO nanocomposite electrode at different current densities. (**d**) Specific capacitance of pristine VOPO_4_, 1:1 VOPO_4_–RGO composite, and 3:1 VOPO_4_–RGO composite as a function of current density; the specific capacitance was calculated from the corresponding discharge curve for each current density. (**e**) EIS curves of 3D VOPO_4_–RGO and 3D VOPO_4_ electrodes obtained in a frequency range of 0.1 Hz to 100 kHz. (**f**) Comparison of capacitance retentions of 3D VOPO_4_–RGO electrode and pristine 3D VOPO_4_ electrode at a scan rate of 100 mV s^−1^.

The electrochemical properties of ice-templated pure 3D VOPO_4_ and the 3D VOPO_4_–RGO nanocomposite were investigated in a three-electrode cell in an aqueous electrolyte of 6 M KOH, with a Pt counter electrode and a Hg/HgO reference electrode. Various mixing ratio of 3D VOPO_4_–RGO nanocomposites were prepared, with VOPO_4_/GO weight ratios of 1:1 and 3:1 (SEM and TEM images in Figure S2). Figure 4a shows the cyclic voltammetry (CV) curves of the 3D-VOPO_4_–RGO (1:1) electrode, 3D-VOPO_4_–RGO (3:1) electrode, and pure-3D-VOPO_4_ electrode at a scan rate of 25 mV s^−1^. We also examined pristine nickel foam, 3D graphene-only and 1:3 VOPO_4_–RGO composite electrodes for comparing electrochemical characteristics of different electrodes (Figure S3). Unlike the pristine nickel foam and 3D graphene-only electrode, a pair of strong anodic and cathodic peaks were observed from the VOPO_4_–RGO composite electrodes which indicate that the capacitive behavior mainly resulted from the pseudocapacitance based on the redox mechanism^33^, and the symmetric features show the excellent reversibility. We propose the following mechanism as the basis of the charge–discharge behavior^23^,^34^,^35^,^36^:


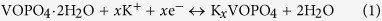


The 3D VOPO_4_–RGO nanocomposite electrodes, especially the one with the 1:1 composition, showed superior electrochemical capacitance, as indicated by higher redox current intensities and larger capacitances compared to those of the 3D VOPO_4_-only electrode. In addition, the shapes of the CV curves barely changed as the scan rates were increased from 2 to 300 mV s^−1^ (Fig. 4b), suggesting fast interfacial charge transfer, excellent electron conduction within the nanosheets, and small interfacial resistance in the nanocomposite electrodes. The galvanostatic charge-discharge curves show symmetric charge and discharge processes even at high current densities (Fig. 4c), indicating the excellent electrochemical features of the 1:1 3D VOPO_4_–RGO nanocomposite as pseudocapacitor electrodes and their superior rate capability. As shown in Fig. 4d, the values of specific capacitance of the 1:1 and 3:1 VOPO_4_–RGO nanocomposite electrodes and the VOPO_4_-only electrode were 527.9, 421.9, and 247.8 F g^−1^ at a current density of 0.5 A g^−1^. This result marks considerably high specific capacitance compared with other 3D graphene/metal oxide approaches^37^,^38^. The maximum capacitance per area is approximately 2.64 F/cm^2^. To understand the reason for the much better capacitive performance of the 3D VOPO_4_–RGO nanocomposite compared to that of pure 3D VOPO_4_, the transport characteristics of the charge carriers within the electrode were investigated using electrochemical impedance spectroscopy (EIS), as shown in Fig. 4e. The diameter of the semicircle on the *Z*_real_ axis is related to the charge-transfer resistance (*R*_ct_). The values of *R*_ct_ of 1:1 VOPO_4_–RGO, 3:1 VOPO_4_–RGO, and pristine VOPO_4_ were 23.5, 29.7, and 131.2 Ω, respectively, representing much improved charge transport in the 3D VOPO_4_–RGO nanocomposites, especially in case of 1:1 weight ratio of electrode. The 3:1 VOPO_4_–RGO composite showed less electrochemical performance because of the shortage of electrically conductive and stable graphene-based framework and increase of self-stacking among less conductive VOPO_4_ nanosheets, as shown in Figure S2.

The much larger specific capacitance of the 1:1 3D VOPO_4_–RGO nanocomposite electrodes compared to that of the 3D VOPO_4_-only electrode can be attributed to the structural uniqueness of VOPO_4_–RGO electrodes^39^,^40^. The 3D VOPO_4_–RGO nanocomposite with interstacked structure could stably hold the VOPO_4_ nanosheets during the pseudocapacitive reaction and allowed most of the VOPO_4_ nanosheets to participate in the reaction as active material. In addition, their maximized plane–plane contact between the VOPO_4_ nanosheets and RGO nanosheets resulted in fluent electron transfer from VOPO_4_ to the highly conductive RGO framework, which cannot be formed in sequential incorporation or synthesis of metal oxides in graphene structures. Moreover, the directionally porous structures formed using the ice-template method significantly increased the accessible surface area and shortened the ion-diffusion length for the electrolyte to increase the specific capacitance of the electrodes. To compare the electrochemical properties with conventional method, 5 wt% of polyvinylidene fluoride (PVDF) binder-containing powder-type VOPO_4_-graphene composite electrode was also examined, as shown in Figure S3. The conventional binder-containing powder method showed similar electrochemical characteristic peaks but the magnitude of electrochemical reaction was much lower than ice-templated VOPO_4_–graphene composite electrode because of the sluggish rate of ion transport during the redox reaction.

The cycle life is also an important issue in the durability of supercapacitor performance. The cycle-life test during 5000 cycles for the 1:1 VOPO_4_–RGO electrode and the VOPO_4_-only electrode was carried out by repeating the CV test between 0 and 0.8 V at a scan rate of 100 mV s^−1^ (Fig. 4f). After 5000 cycles, the capacitance retention of the 3D VOPO_4_-only electrode was ~53%, while the capacitance of the 3D VOPO_4_–RGO nanocomposite electrode can maintain ~85% of the initial value, revealing excellent stability. In general, low-dimensional materials usually exhibit the tendency to lose the initial capacitance during cycling because of agglomeration and the corresponding decreased surface area when exposed to the electrolyte^41^. However, the stable 3D network structure of the VOPO_4_–RGO nanocomposite effectively suppressed agglomeration of VOPO_4_ and thus, long-term charge storage was realized. It is worth noting that the specific capacitance increased during the initial 300 cycles, which was probably related to an improvement in the surface wetting of the interstacked electrode by the initial charge–discharge process and further activation of the shielded VOPO_4_ active material^42^. All these results reveal that the interconnected 3D framework of the nanocomposite was an ideal ordered structure for a supercapacitor electrode.

**Figure 5:**
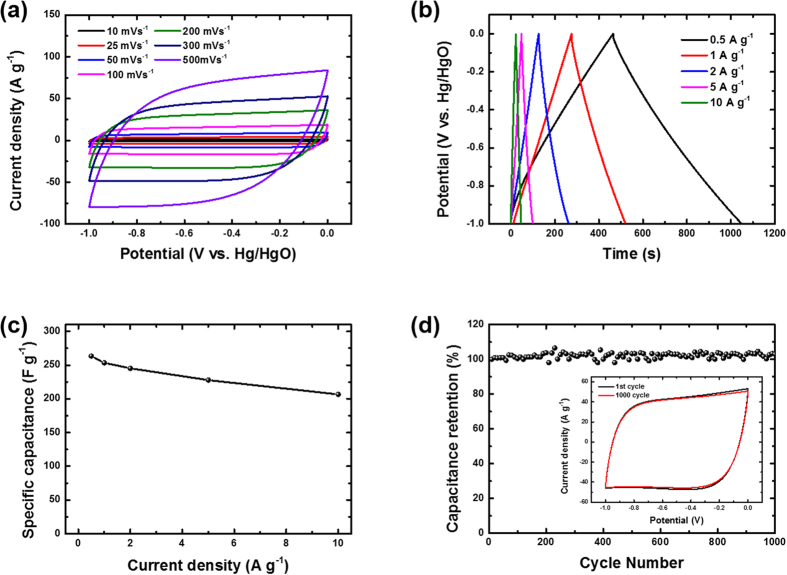
(**a**) CV curves of vertically porous 3D graphene at different scan rates in 6 M KOH. (**b**) Galvanostatic charge–discharge curves of porous 3D graphene at different constant current densities. (**c**) Specific capacitance of porous 3D graphene as a function the current density; the specific capacitance was calculated from the corresponding discharge curve for each current density. (**d**) Cycle performance of the porous 3D graphene electrode at a scan rate of 300 mV s^−1^ over 1000 cycles.

Asymmetric supercapacitor systems that incorporate different materials for each electrode promise a wider operating voltage and thus provide increased energy densities^43^. To explore the advantage of the interconnected 3D framework of the VOPO_4_–RGO nanocomposite for practical applications, we fabricated an ASC using the 3D VOPO_4_–RGO nanocomposite as the positive electrode and the vertically porous 3D RGO prepared by the same ice-templating method as the negative electrode. The electrochemical performance of the vertically porous 3D RGO electrode is shown in Fig. 5. Owing to its high conductivity and vertically porous structure, the rectangular shapes of the CV curves at scan rates of 10–500 mV s^−1^ generally remained unchanged (Fig. 5a), indicating excellent double-layer capacitive behavior and low internal resistance at the RGO–nickel foam interface^44^. The linear profile of galvanostatic charge and discharge curves and their symmetric triangular shape remained consistent, which also represents typical EDLC behavior and good reversibility (Fig. 5b)^45^. The specific capacitances of the vertically porous 3D RGO electrode estimated from the discharging curves were 263.4–206.6 F g^−1^ at current densities of 0.5–10 A g^−1^, indicating the good rate capability of the porous 3D graphene (Fig. 5c). Moreover, our vertically porous 3D graphene electrode exhibited an excellent long cycle life without noticeable decrease after 1000 cycles (Fig. 5d).

**Figure 6:**
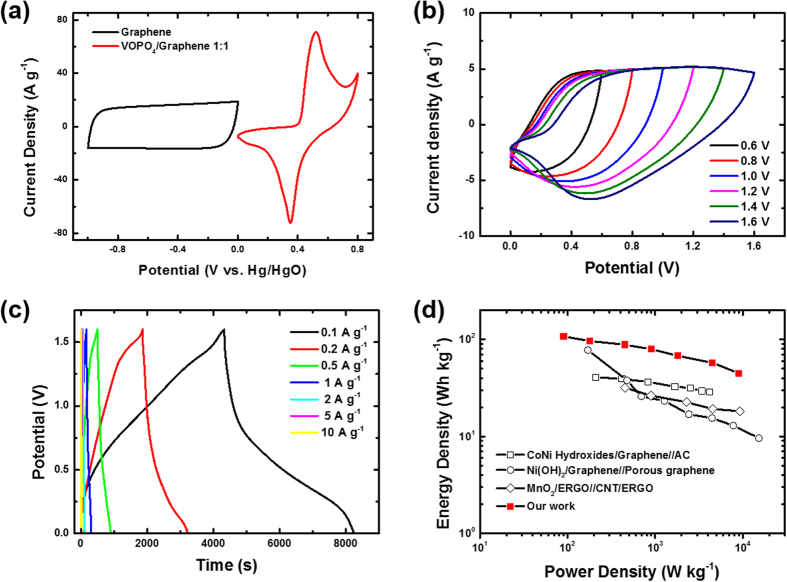
(**a**) Comparative CV curves of vertically porous 3D graphene electrode and 3D VOPO_4_–RGO electrode obtained in three-electrode setup in 6 M KOH electrolyte at a scan rate of 50 mV s^−1^. (**b**) CV curves of VP3D-VOPO_4_–RGO/VP3D-RGO asymmetric supercapacitor (ASC) in different operation voltages from 0.6 to 1.6 V at a scan rate of 50 mV s^−1^. (**c**) Galvanostatic charge–discharge curves of ASC at different constant current densities from 0.1 to 10 A g^−1^. (**d**) Ragone plot (energy density vs. power density) of VP3D-VOPO_4_–RGO/VP3D-RGO ASC and other ASCs recently reported in the literature.

We compared the CV curves of the 3D VOPO_4_–RGO electrode and the vertically porous 3D RGO electrode in 6 M KOH electrolyte, obtained in a three-electrode cell at a scan rate of 50 mV s^−1^, and the results are shown in Fig. 6a. It can be clearly seen that the sum of the potential ranges of these two electrodes was 1.6 V, indicating that they could potentially be used in a high-voltage asymmetric supercapacitor. Thus, an asymmetric supercapacitor was fabricated using VOPO_4_–RGO as the positive electrode and the vertically porous 3D RGO as the negative electrode, with the charge flow balanced between the positive and negative electrodes. Based on the CV analysis, we confirmed that the 3D VOPO_4_–RGO nanocomposite electrode and the vertically porous 3D RGO electrode had a stable operating cell voltage that could be extended to 1.6 V. (Fig. 6b) The CV curves of the optimized VP3D-VOPO_4_–RGO/VP3D-RGO ASC at various scan rates showed almost no significant change even at a high scan rate of 100 mV s^−1^ (Figure S4a). This result implies that the redox reaction was not kinetically limited at least within the scan rates of 2–100 mV s^−1^, which indicates an improved rate capability^46^. In galvanostatic charge–discharge experiments in a voltage window of 0–1.6 V, a region of non-linearity in the discharge curves at lower current density was observed, but the charging and discharging curves were still nearly symmetric, confirming the highly reversible electrochemical charge storage (Fig. 6c)^47^. The specific capacitance of the VP3D-VOPO_4_–RGO/VP3D-RGO ASC reached 336.7 F g^−1^ at a current density of 0.1 A g^−1^ while still retaining a value of 140.3 F g^−1^ at a higher current density of 10 A g^−1^ (Figure S4b).

The energy and power densities of the asymmetric supercapacitor were calculated from the galvanostatic discharge curves and plotted on the Ragone diagram shown in Fig. 6d. The VP3D-VOPO_4_–RGO/VP3D-RGO ASC with a cell voltage of 1.6 V exhibited an energy density of 108 Wh kg^−1^. This hybridized nanostructured ASC also exhibited much higher energy density than other metal-oxide-based ASCs that have been reported recently, as summarized in Table S1^39^,^48^,^49^. The energy density of the asymmetric capacitor was significantly improved because of the high specific capacitance of the electrodes and the wide operation voltage window. Furthermore, the vertically porous 3D graphene in both electrodes demonstrated their distinctive advantages for asymmetric supercapacitors. Because of the graphene nanosheets’ excellent mechanical properties, good electrochemical stability, and excellent conductivity, in addition to acting as the support for the 2D VOPO_4_, they also maintained the overall mechanical integrity and high electrical conductivity of the materials for fast redox reactions.

The vertically porous hybrid structure consisting of the 3D VOPO_4_–RGO nanocomposite supported on macroporous nickel foam was successfully fabricated using a simple ice-template self-assembly technique. The resulting 3D VOPO_4_–RGO structure had high surface area, aligned and directionally porous structure, and high electrical conductivity, facilitating the diffusion of electrolytes and fast transportation of charge carriers within the porous framework. The obtained electrodes exhibited impressive electrochemical performance with better cycling stability. An asymmetric supercapacitor based on 3D VOPO_4_–RGO as the positive electrode and porous 3D RGO as the negative electrode, delivered a high energy density of up to 108 Wh kg^−1^. In addition, the energy density of the as-obtained asymmetric supercapacitor significantly exceeded those of most recently reported asymmetric supercapacitors using other electrode materials.

## Methods

### Preparation of samples

VOPO_4_ was obtained according to a simple method reported in the previous literature^29^. The mixture, including V_2_O_5_ (4.8 g, Aldrich), H_3_PO_4_ (85% 26.6 ml, Aldrich) and H_2_O (115.4 ml), was refluxed at 110 °C for 24 h. After cooling naturally, the yellow precipitates were collected by centrifugation, washed with de-ionized water and acetone, and dried in a vacuum oven at 60 °C for overnight. VOPO_4_ nanosheets were fabricated by liquid exfoliation of bulk VOPO_4_ powders via a sonication in isopropanol. The sealed flask was sonicated for 3 h, and then the dispersion was centrifuged at 2000 rpm for 10 min to remove aggregates. GO used in this work was synthesized by a modified Hummers method^30^. Vertically porous 3D hybrid structures were synthesized by freeze casting and chemical reduction process. Typically, a 10 mL of GO (6.0 mg mL^−1^) aqueous dispersion were mixed with 5 mL of isopropanol suspension of VOPO_4_ nanosheets with different concentrations by sonication for 2 h. Then, nickel foam (1.5 cm × 1 cm × 0.1 cm, 100 mg, from Wellcos co. Ltd.) was immersed into the above stable dispersions, followed by sonication for 1 h and then placed under vacuum to assist in the removal of air bubbles. The dispersion was aging for 24 h at 60 °C. Afterward, the nickel foam filled with suspensions were fabricated using ice-templated method. Briefly, the solution was poured into a glass tube was unidirectionally frozen by liquid nitrogen, and frozen entirely. The frozen samples were freeze-dried for 3 days under a vacuum to form hybrid aerogels within nickel foam. The VOPO_4_–GO or GO electrodes were reduced by hydrazine vapor at 80 °C overnight.

### Structural characterization

The structural morphology and the in-detail inter-stacked structure of the samples was examined by FE-SEM (JSM 6701F, JEOL) and TEM (FEI Tecnai G2, PHILIPS). Atomic force microscopy (AFM) images were obtained using MFP3D microscope (Asylum Research). For the preparation of TEM and AFM samples, the powder sample was sonicated in ethanol for 5 min and the suspension was dropped on a Cu grid for TEM and on the freshly carved mica for AFM sample. The crystal structures of the materials were determined by a Rigaku XRD system equipped with Cu K α radiation (λ = 0.15406 nm). Pore structure of the samples was characterized by physical adsorption of N_2_ at 77 K using a BELSORP-max nitrogen adsorption apparatus (Japan Inc.). The specific surface area was calculated with Brunauer-Emmett-Teller (BET) method from the N_2_ adsorption isotherm. The electrical conductivity of the samples was measured using the four-point probe method (Keithley 2400) using pelletized samples.

### Electrochemical measurements

The electrochemical tests of the individual electrode were performed in a three electrode cell, in which Pt mesh and Hg/HgO electrode was used as the counter and reference electrodes, respectively. The electrochemical properties of asymmetric supercapacitor were investigated under a coin-type cell (CR2032) with 3D VOPO_4_–RGO as the positive electrode, a polypropylene separator (Celgard 3501) and porous 3D RGO as the negative electrode in 6 M KOH electrolyte solution. To fabricate an asymmetric supercapacitor, the loading mass ratio of active material (VP3D VOPO_4_–RGO: VP3D RGO) was estimated to be 0.63 from the specific capacitance from their galvanostatic charge–discharge curves. The performances for both three-electrode and two-electrode configurations were measured with Autolab PGSTAT-128N instrument (Eco-chemie). The electrochemical impedance spectroscopy (EIS) were measurement was performed by applying an AC voltage with 5 mV amplitude in a frequency range 0.01–100 kHz at open circuit voltage.

The specific capacitance values of a single electrode was calculated from galvanostatic charge-discharge curves as follows: C_sp_ = (I × ∆t)/(∆V × m), where C_sp_ is the specific capacitance (F g^−1^) based on the mass of the active materials, I is discharge current (A), ∆t is discharge time (s), ∆V is potential change during the discharge (V), and m (g) is the mass of the active materials in the electrode. The specific capacitance of asymmetric supercapacitor was calculated from galvanostatic charge-discharge curves as follows: C_sp_ = (I × ∆t)/(∆V × m), where m (g) is the total mass of the active material in the positive and negative electrodes. The energy and power densities of the asymmetric supercapacitor were calculated as follows: E = 1/2 × C × (∆V^2) and P = E/∆t, where E (Wh kg^−1^) is the energy density, ∆V (V) is potential change during the discharge, P (W kg^−1^) is the power density, and ∆t (s) is the discharge time.

## Additional Information

**How to cite this article**: Lee, K. H. *et al.* Ice-templated Self-assembly of VOPO_4_–Graphene Nanocomposites for Vertically Porous 3D Supercapacitor Electrodes. *Sci. Rep.* **5**, 13696; doi: 10.1038/srep13696 (2015).

## Supplementary Material

Supplementary Information
