# Supplementary material for: Ice-templated Self-assembly of VOPO4–Graphene Nanocomposites for Vertically Porous 3D Supercapacitor Electrodes
Source: Sci Rep. 2015 Sep 3;5:13696. doi: 10.1038/srep13696 (PMC4558581; doi:10.1038/srep13696)
Supplement: Supplementary Information [file srep13696-s1.pdf]

Supplementary Information

Ice-templated Self-assembly of $VOPO_4$–Graphene Nanocomposites for Vertically Porous 3D Supercapacitor Electrodes

*Kwang-Hoon Lee, Young-Woo Lee, Seung Woo Lee, Jeong Sook Ha, Sang-Soo Lee, and Jeong Gon Son**

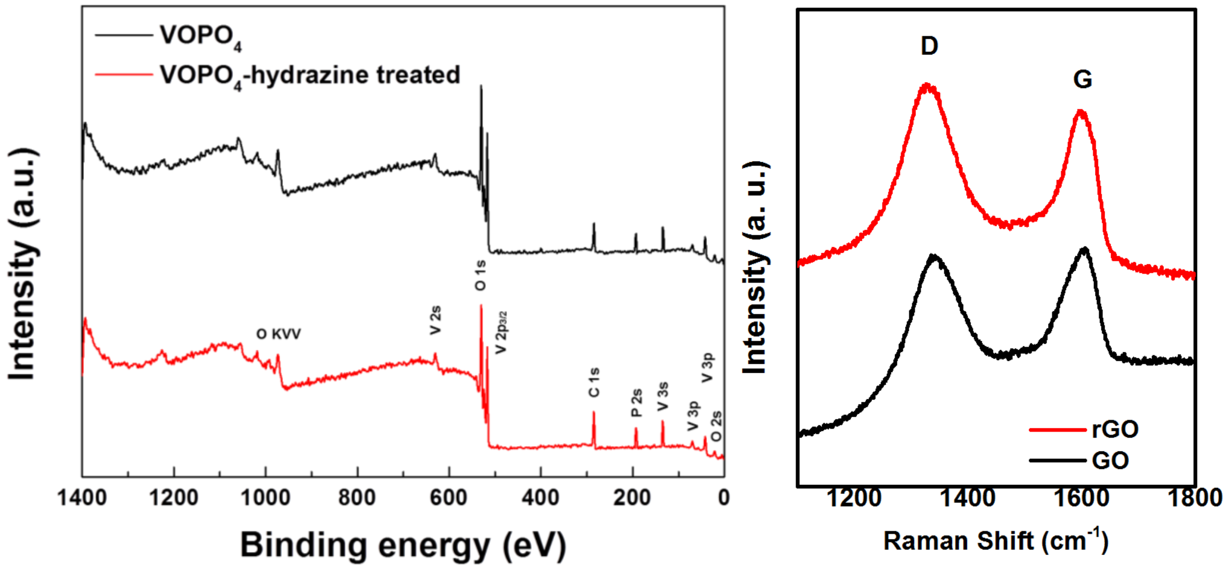

Figure S1. (a) XPS spectra for $VOPO_4$ nanosheet and (b) Raman spectrum of graphene oxide (GO) and reduced rGO before and after hydrazine vapor treatment.

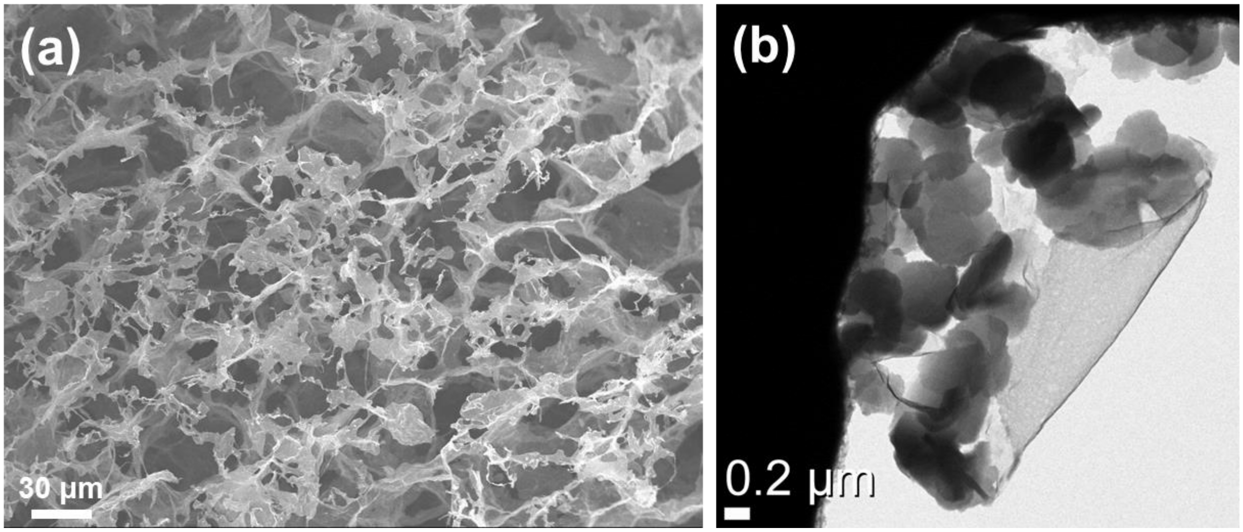

Figure S2. (a) SEM and (b) TEM images of $VOPO_4$–RGO nanocomposites with 3:1 weight ratio between $VOPO_4$ and RGO, respectively.

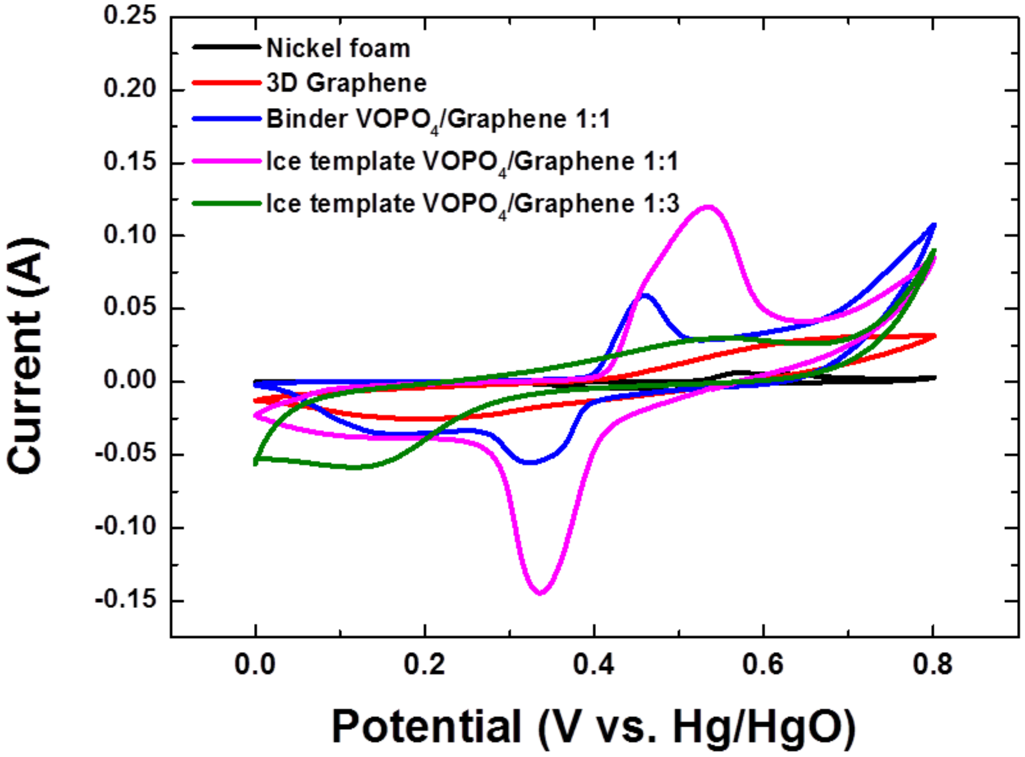

Figure S3. CV curves of pristine nickel foam, vertically porous 3D graphene, 5 wt% PVDF binder-containing powder $VOPO_4$–RGO nanocomposite with 1:1 weight ratio, ice-templated vertically porous 3D $VOPO_4$–RGO nanocomposites with 1:1 and 1:3 weight ratios of positive electrodes (3 mg) in 100 mg of nickel foam at a scan rate of 25 mV s^{-1} in 6 M KOH electrolyte.

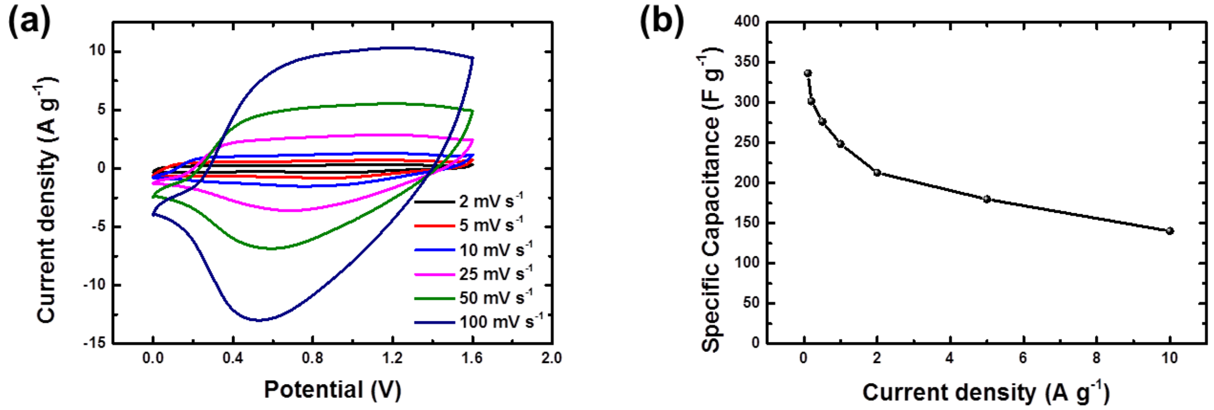

Figure S4. (a) CV curves of VP3D-$VOPO_4$–RGO/VP3D-RGO ASC at different scan rates in the voltage window of 0–1.6 V. (b) Specific capacitance of ASC as a function the current densities calculated from the corresponding discharge curve for each current density.

Table S1. Performances comparison of ASCs with previously reported ASCs in aqueous electrolytes.

ASC structure	Electrolyte	Operation Voltage (V)	Energy density (Wh kg^{-1})	Ref.
**$GrMnO_2/GrMoO_3$**	Na_2SO_4	2.0	42.6	**[S1]**
**RGO-RuO_2/RGO-PANI**	KOH	1.4	26.3	**[S2]**
**$Ni(OH)_2$-UGF/a-MEGO**	KOH	1.8	13.4	**[S3]**
**CoO@PPy/AC**	NaOH	1.8	43.5	**[S4]**
**CoNi Hydroxides-Graphene/AC**	KOH	1.4	40.8	**[S5]**
**$Ni(OH)_2$-graphene/porous graphene**	KOH	1.6	77.8	**[S6]**
**MnO_2-ERGO/CNT-ERGO**	PAAK/KCl	1.8	31.8	**[S7]**
**$Co(OH)_2$ nanoflakes/AC**	KOH	1.6	92.7	**[S8]**
**VP3D-$VOPO_4$-RGO/VP3D-RGO**	**KOH**	**1.6**	**108**	**Our work**

References

[S1] J. Chang, M. Jin, F. Yao, T. H. Kim, V. T. Le, H. Yue, F. Gunes, B. Li , A. Ghosh, S. Xie and Y. H. Lee, *Adv. Funct. Mater.*, 2013, **23**, 5074-5083.

[S2] J. Zhang, J. Jiang, H. Li and X. S. Zhao, *Energy Environ. Sci.*, 2011, **4**, 4009-4015.

[S3] J. Ji, L. Zhang, H. Ji, Y. Li, X. Zhao, X. Bai, X. Fan, F. Zhang and R. S. Ruoff, *ACS Nano* 2013, **7**, 6237-6243.

[S4] C. Zhou, Y. Zhang, Y. Li and J. Liu, *Nano lett.*, 2013, **13**, 2078-2085.

[S5] Y. Cheng, H. Zhang, C. V Varanasi, J. Liu, *Energy Environ. Sci.* 2013, **6**, 3314.

[S6] J. Yan, Z. Fan, W. Sun, G. Ning, T. Wei, Q. Zhang, R. Zhang, L. Zhi and F. Wei, *Adv. Funct. Mater.*, 2012, **22**, 2632-2641.

[S7] Z. Zhang, F. Xiao, L. Qian, J. Xiao, S. Wang, Y. Liu, *Adv. Energy Mater.* **2014**, *4*, 10.1002/aenm.201400064.

[S8] L.-B. Kong, M. Liu, J.-W. Lang, Y.-C. Luo, L. Kang, *J. Electrochem. Soc.* **2009**, *156*, A1000.
